# Extensive Basal Level Activation of Complement Mannose-Binding Lectin-Associated Serine Protease-3: Kinetic Modeling of Lectin Pathway Activation Provides Possible Mechanism

**DOI:** 10.3389/fimmu.2017.01821

**Published:** 2017-12-18

**Authors:** Gábor Oroszlán, Ráhel Dani, András Szilágyi, Péter Závodszky, Steffen Thiel, Péter Gál, József Dobó

**Affiliations:** ^1^Institute of Enzymology, Research Centre for Natural Sciences, Hungarian Academy of Sciences, Budapest, Hungary; ^2^Department of Biomedicine, Aarhus University, Aarhus, Denmark

**Keywords:** innate immunity, complement, lectin pathway, serine protease, proenzyme, autoactivation, reaction kinetics

## Abstract

Serine proteases (SPs) are typically synthesized as precursors, termed proenzymes or zymogens, and the fully active form is produced *via* limited proteolysis by another protease or by autoactivation. The lectin pathway of the complement system is initiated by mannose-binding lectin (MBL)-associated SPs (MASP)-1, and MASP-2, which are known to be present as proenzymes in blood. The third SP of the lectin pathway, MASP-3, was recently shown to be the major activator, and the exclusive “resting blood” activator of profactor D, producing factor D, the initiator protease of the alternative pathway. Because only activated MASP-3 is capable of carrying out this cleavage, it was presumed that a significant fraction of MASP-3 must be present in the active form in resting blood. Here, we aimed to detect active MASP-3 in the blood by a more direct technique and to quantitate the active to zymogen ratio. First, MASPs were partially purified (enriched) from human plasma samples by affinity chromatography using immobilized MBL in the presence of inhibitors. Using this MASP pool, only the zymogen form of MASP-1 was detected by Western blot, whereas over 70% MASP-3 was in an activated form in the same samples. Furthermore, the active to zymogen ratio of MASP-3 showed little individual variation. It is enigmatic how MASP-3, which is not able to autoactivate, is present mostly as an active enzyme, whereas MASP-1, which has a potent autoactivation capability, is predominantly proenzymic in resting blood. In an attempt to explain this phenomenon, we modeled the basal level fluid-phase activation of lectin pathway proteases and their subsequent inactivation by C1 inhibitor and antithrombin using available and newly determined kinetic constants. The model can explain extensive MASP-3 activation only if we assume efficient intracomplex activation of MASP-3 by zymogen MASP-1. On the other hand, the model is in good agreement with the fact that MASP-1 and -2 are predominantly proenzymic and some of them is present in the form of inactive serpin–protease complexes. As an alternative hypothesis, MASP-3 activation by proprotein convertases is also discussed.

## Introduction

The complement system, as an essential part of the innate immune response, eliminates invading microorganisms and dangerous host cells (1, 2). The complement cascade is composed of more than 30 proteins. Key components of the system are serine proteases (SPs), which typically circulate in bloodstream in the zymogen form until their successive cleavage and activation (3). Complement activation can be triggered *via* three different, however interconnected routes: the classical, lectin, and alternative pathways, then the three routes converge into the common terminal pathway. When the classical or lectin pathways are activated, it results in the formation of the C3 convertase, C4bC2a, composed of the cleaved forms of complement factors C4 and C2 (4). The alternative pathway serves as an amplification loop, but it can also be activated on its own by the “tick-over” mechanism (5).

Activation of the lectin pathway is initiated on surfaces displaying various arrays of carbohydrates or acetyl groups, which can be recognized by (at least) five different pattern recognition molecules (PRMs): mannose-binding lectin (MBL), H-ficolin (ficolin-3), L-ficolin (ficolin-2), M-ficolin (ficolin-1), and CL-LK (a heterocomplex of collectin liver 1 and collectin kidney 1) (6–8). The recognition of dangerous patterns is transformed to enzymatic signals by two proteases complexed with the PRMs, MBL-associated SP 1 and 2 (MASP-1, MASP-2). The first proteolytic step is the autoactivation of MASP-1, which then activates MASP-2. Both enzymes can cleave C2, however C4 is cleaved only by MASP-2. As a consequence both MASP-1 and MASP-2 play essential roles in the formation of the C4bC2a enzymatic complex (9–11).

All MASPs have the same six-domain structure (4, 7). The C-terminal SP domain is preceded by five regulatory domains in the order of CUB1-EGF-CUB2-CCP1-CCP2. CUB stands for C1r/C1s, sea urchin Uegf and bone morphogenetic protein-1 domain, EGF for epidermal growth factor domain, and CCP for complement control protein domain. Upon activation an Arg-Ile (R-I) bond is cleaved within the SP domain and the resulting two chains, termed A and B, are held together by a disulfide bridge.

Mannose-binding lectin-associated SP-1 has relatively broad substrate specificity (12), as a result it is involved in coagulation (13) and certain proinflammatory reactions, e.g., cleavage of PAR receptors on endothelial cells results in their activation, and cleavage of high molecular weight kininogen produces the proinflammatory bradykinin (4, 14, 15). The role of two non-catalytic associated proteins (MAp19 or sMAP, MAp44, or MAP1) is less clear (16–19). The third SP of the lectin pathway, MASP-3 (20), along with MAp19 and MAp44 were initially considered simply as negative lectin pathway regulators, but recent results implicated that MASP-3 has an important role in the activation of the complement system in connection with the alternative pathway (21–25).

Factor D (FD), a single-domain SP, is a key enzyme for the alternative pathway. FD has only one natural substrate, factor B (FB) complexed with C3b (26). Until recently, it was believed that FD is activated at the site of the synthesis, maybe even before secretion (27), because only active FD could be purified from normal blood (28, 29). Although in mammalian cell cultures predominantly active FD was detected (30, 31), the zymogen form was also present (31). In insect cells, proenzyme FD (pro-FD) is expressed almost exclusively (22, 32). Recent results by Pihl et al. (25) and personal discussions with Elod Kortvely (unpublished data) suggest that both FD and pro-FD are present in normal human blood with FD being the dominant form.

A link between early complement pathways was proposed by Takahashi et al. observing the lack of alternative pathway activity in *MASP1* knock-out mice. Due to the fact that MASP-1 and MASP-3 are two splice variants from the same gene such mice lacks both proteins. In the absence of both MASP-1 and MASP-3 only zymogen FD was detected. At first, MASP-1 was suggested to be responsible for pro-FD activation, although supplementing the serum from such mice with recombinant MASP-1 did not restore the alternative pathway (33). Later, MASP-3 was also implicated as an enzyme capable of converting pro-FD to active FD, and even proenzyme MASP-3 was proposed to be able to carry out such cleavage (21). Subsequent results questioned the involvement of MASP-1 and/or MASP-3 in alternative pathway activation. In the serum of a MASP-1/3-deficient patient, suffering from the Malpuech–Michels–Mingarelli–Carnevale (3MC) syndrome causing serious craniofacial defects, functional alternative pathway activity was observed (10). Nevertheless a following discussion revealed that mainly pro-FD can be detected in the blood of these 3MC patients (34). Furthermore, uncontrolled alternative pathway activity was observed in MASP-1/3 and factor H codeficient mice just like in knock-out mice deficient for factor H only (35). These data contradicted the view that MASP-1 and/or MASP-3 is involved in the function of the alternative pathway, and the exact way of pro-FD conversion remained unclear.

Our previous studies clarified this controversy and the role of MASPs in pro-FD activation. First, we determined the rate constants of pro-FD cleavage for all three MASPs. We found that only the active forms of MASPs are able to convert pro-FD to FD. Next, using fluorescently labeled recombinant pro-FD, we detected the activation of this exogenous pro-FD in human blood samples. With our previously developed MASP-1- and MASP-2-specific inhibitors we ruled out MASP-1 and MASP-2 as potential pro-FD activators in resting blood (22). Then, using a MASP-3-specific inhibitor, we showed that MASP-3 is the major activator of pro-FD in general, and it is the exclusive pro-FD activator under resting conditions in human blood (23). By “resting” conditions, we mean that neither the complement nor the coagulation system is activated above the basal level. Furthermore, our results supported thrombin and/or another coagulation protease serving as a potential backup enzyme for this conversion in coagulated blood (i.e., human serum) (22, 23). Our findings also imply that under resting conditions, at least a fraction of MASP-3 must be present in the active form in the bloodstream (22, 23). The average serum or plasma concentration of MASP-3 was published in several studies as about 5–7 µg/ml (36–39), however, the extent of MASP-3 activation has remained unknown.

In this study, we aimed to determine the active to zymogen MASP-3 ratio in normal resting human blood. In order to minimize activation during sample preparation, we used EDTA plasma (pooled or individual) as starting material, then MASPs were purified on MBL-Sepharose in the presence of broad specificity inhibitors, Pefabloc and 4-nitrophenyl 4′-guanidinobenzoate (NPGB). MASPs were then detected by Western blotting. Our data imply that MASP-3 circulates mostly in the active form in human blood. Of course the question arises how MASP-3 is activated, especially in the light of the fact that MASP-3, unlike MASP-1, cannot autoactivate. In an attempt to explain the observed facts, a kinetic model of lectin pathway activation was set up, which, using reasonable assumptions, provides a solution for the phenomenon.

## Materials and Methods

### Proteins, Reagents, Blood Samples, and Antibodies

Active recombinant MASP-1 and MASP-2 catalytic fragments (MASP-1cf, MASP-2cf), encompassing the complement control protein domains 1 and 2 and the SP domain (CCP1-CCP2-SP), were produced as described (11, 40, 41). Stable zymogen mutant forms MASP-1cf (R448Q) and MASP-2cf (R444Q) were prepared as described (11, 42). Zymogen MASP-3cf (also the CCP1-CCP2-SP region) was produced and then further purified as described (11, 22). Activated MASP-3cf was prepared from zymogen MASP-3cf, and then further purified as described (22, 43). Pro-FD was produced in insect cells and then it was activated as described (22). CNBr-Activated Sepharose 4 Fast Flow resin was purchased from GE Healthcare. Recombinant human mannan-binding lectin (rMBL), produced based on the protocol of Vorup-Jensen et al. (44), was from Enzon Pharmaceuticals.

Blood was drawn from ten healthy volunteers into S-Monovette K3E EDTA tubes (Sarstedt) to produce fresh pooled plasma. After centrifugation, samples were combined, and kept at −70°C in aliquots. Blood was drawn from seven healthy volunteers into S-Monovette K3E EDTA tubes (Sarstedt) to produce fresh individual plasma samples. After centrifugation, samples were kept at −70°C in aliquots. The study was conducted in conformity with the WMA Declaration of Helsinki. Experimental protocols were approved by the local ethics committee (permission number: TUKEB 9190-1/2017/EKU). Informed consent was obtained for the isolation of peripheral venous blood from the donors.

Pefabloc SC was purchased from Sigma-Aldrich (Fluka brand). NPGB was purchased from Merck. Monoclonal antibody against human complement MASP-3 catalytic domain was purchased from RD Systems (catalog number: MAB1724). Anti-Mouse IgG alkaline phosphatase antibody (catalog number A2429), 4-nitro blue tetrazolium chloride (NBT), and the 5-bromo-4-chloro-3-indolyl phosphate disodium salt (BCIP) were purchased from Sigma-Aldrich. The Precision Plus Protein Kaleidoscope Prestained Standard was from Bio-Rad, the LMW-SDS Marker was from GE Healthcare.

### MASP-1-SP, Anti-MASP-1-SP Polyclonal Antibody, and Its Alkaline Phosphatase Conjugate

The MASP-1-SP (MASP-1 SP domain) gene was amplified from the MASP-1cf construct (41). The forward primer (5′-GGCC***GCTAGC***ATGACTGTGTGTGGGCTCCCCAAG-3′) contained an expression enhancing sequence (Ala-Ser-Met-Thr, underlined) and a NheI restriction site (bold and italic), while the reverse primer (5′-CG***GAATTC***TCAGTTCCTCACTCCGGT-3′) contained an EcoRI restriction site (bold and italic). The NheI-EcoRI fragment of the PCR product was ligated into a pET17b plasmid (Novagen). The pET17b_MASP-1-SP construct was transformed into *Escherichia coli* BL21 (DE3) pLysS host strain (Novagen) and the clones were selected in presence of ampicillin and chloramphenicol (SERVA). The expressed MASP-1-SP formed inclusion bodies; the pellet was collected and solubilized as described (40). The solubilized protein was added at 100 mg/L final concentration into refolding buffer containing 50 mM Tris, 0.5 M arginine, 0.5 M NaCl, 5 mM EDTA, 6 mM glutathione, 4 mM oxidized glutathione, pH 8.0. The refolding solution was incubated at 4°C for 2–4 weeks then dialyzed against 10 mM Na_2_HPO_4_, 0.5 mM EDTA, pH 6.8 buffer, and filtrated on 0.45 µm cellulose-acetate membrane (Sartorius Stedim biotech). MASP-1-SP was loaded to a 26-mm × 100-mm SP Sepharose High Performance (GE Healthcare) column equilibrated with 10 mM Na-phosphate, pH 6.8 and eluted with a 0–200 mM NaCl linear gradient in the same buffer. Selected fractions were combined and diluted 10-fold in 10 mM Tris, pH 8.2. The sample was applied to a 16-mm × 100-mm YMC BioPro Q30 (YMC Europe) column equilibrated with the same buffer, and eluted with a 0–300 mM NaCl linear gradient. Several MASP-1-SP batches were pooled, dialyzed against 10 mM Na_2_HPO_4_, 150 mM NaCl, pH 7.4, concentrated to 1 mg/ml using spin concentrators, then passed through sterile syringe filter units.

Two rabbits were immunized by subcutaneous injections of MASP-1-SP. The first dose was 0.4 mg MASP-1-SP with Freund’s complete adjuvant. After 4 weeks, the injections were repeated four times, once in every fortnight with 0.2 mg MASP-1-SP in Freund’s incomplete adjuvant. Preimmune blood was withdrawn before the treatment and test blood samples were withdrawn a week after every boost from ear vein and finally the rabbits were bled after sixteen weeks. Sera were obtained by coagulation and centrifugation of the blood samples.

A two-step affinity chromatography purification method was used to obtain MASP-1-SP-specific antibodies from the pooled antiserum of two rabbits. Whole IgG was purified from the 10-fold diluted immunsera using a 10 mm × 100 mm Protein A (MabSelect SuRe, GE Healthcare) column. The sample was loaded in 10 mM Na_2_HPO_4_, 1 M NaCl, 1 mM EDTA, pH 7.0 buffer, then eluted with a linear pH gradient ending in 100 mM citrate, pH 3.0. The immunoglobulin fractions were combined and neutralized with 1 M Tris base, then further purified on MASP-1cf (R448Q)-Sepharose resin. To produce the resin, 1 ml rehydrated CNBr-Activated Sepharose 4 Fast Flow (GE Healthcare) resin was reacted with 6 mg MASP-1cf (R448Q) according to the manufacturers protocol. The suggested final washes with solutions of alternating pH were omitted. The pooled IgG fraction was twofold diluted with 20 mM Tris, 150 mM NaCl, 0.3% Tween-20, pH 7.5 buffer and applied to a 5 mm × 50 mm MASP-1cf (R448Q)-Sepharose column. Two overlapping populations of anti-MASP-1-SP antibodies, weakly and strongly binding, were eluted with linear pH gradient ending in 100 mM citrate, pH 3.0. The fractions were neutralized with 1 M Tris base and treated with 0.02% NaN_3_ as preservative. Part of the strongly binding population of anti-MASP-1-SP antibodies was concentrated to 1 mg/ml and conjugated with alkaline phosphatase enzyme (AP). The conjugation was performed by Alkaline Phosphatase Labeling Kit-NH_2_ (Abnova) using the manufacturers protocol.

### MASP Purification from EDTA Plasma on MBL-Sepharose

Mannose-binding lectin-Sepharose had been previously used by others to purify recombinant MASPs (45, 46). We modified this method to purify MASPs (and MAPs) from human EDTA plasma. Recombinant human MBL was dialyzed excessively against coupling buffer (100 mM NaHCO_3_, 500 mM NaCl, pH 8.3), then concentrated to 4.75 mg/ml with 10 kDa cutoff concentrator. The concentration of rMBL was calculated based on the extinction coefficient of ε_280_ = 18,365 M^–1^ cm^–1^ and a molecular mass of a polypeptide of 24.0 kDa (not accounting for glycosylation).

Half a gram of CNBr-Activated Sepharose 4 Fast Flow resin was washed with 50 ml 1 mM HCl solution, then with sterile water, and finally equilibrated with coupling buffer. The resin was mixed with 2.1 ml 4.75 mg/ml rMBL and the mixture was rotated gently for 4 h at room temperature, and subsequently the beads were washed with 10 ml coupling buffer. After coupling the resin was washed with 5 ml 100 mM Tris (pH 8.0) buffer, then incubated for overnight with 8 ml of the same buffer at 4°C, then washed with 150 mM NaCl, 50 mM Tris, 10 mM CaCl_2_ (pH 7.5) buffer and stored in the same buffer containing 0.02% NaN_3_. The coupling efficiency was about 2.5 mg rMBL per ml wet resin.

Plasma, 5 ml, containing about 10 mM EDTA (pool of 10 donors or individual) was thawed at room temperature, and then was immediately mixed with 65 µl inhibitor stock solution [10 mM NPGB, 100 mM Pefabloc in anhydrous DMF (Merck)]. NaCl, 1.25 ml of 5 M, and 315 µl of 1 M Tris (pH 8.0) solutions were added to plasma containing the inhibitors, then supplemented with 100 µl of MBL-Sepharose resin. The mixture was rotated for 30 min at 0–8°C, then further 65 µl inhibitor stock solution was added. After twofold dilution with ice-cold sterile water, 85 µl of 2 M CaCl_2_ (12.5 mM final) was added, then it was rotated for 30 min at 4°C. Further steps were performed at room temperature however using ice-chilled buffers. The mixture containing the resin was loaded to a Poly-Prep Chromatography column (Bio-Rad, catalog number: 731-1550). The flow-through was discarded, the resin was washed with 500 µl ice-cold 50 mM Tris, 150 mM NaCl, 10 mM CaCl_2_, 0.1 mM NPGB, 1 mM Pefabloc (pH 7.5) buffer, then with 200 µl of ice-cold 50 mM Tris, 150 mM NaCl, 10 mM EDTA, 0.1 mM NPGB, 1 mM Pefabloc, pH 7.5 buffer. After the washing steps an end cap was applied to column, and the resin was incubated with 150 µl elution buffer (50 mM Tris, 1 M NaCl, 20 mM EDTA, 0.1 mM NPGB, 1 mM Pefabloc, pH 8.0) for 5 min. Following incubation the end cap was removed and the eluate was collected, then another 150 µl of elution buffer was applied to column. The two eluted fractions were combined and after twofold dilution with ice-cold sterile water, the sample was mixed with 1/3 volume of 4× SDS-PAGE sample buffer then heated for 2 min at 95°C.

In order to produce samples without inhibitors, the same experiment was performed, but the last washing and the elution buffers did not contain NPGB and Pefabloc. After elution and dilution the mixture was incubated with MASP-1cf at 215 nM final concentration at 37°C, and aliquots were removed at 1.5 and 5 h. The reaction was stopped by adding 1/3 volume of 4× SDS-PAGE sample buffer and heating for 2 min at 95°C.

### Western Blot Analysis

Proteins in the samples were separated by SDS-PAGE under both reducing and non-reducing conditions. Stacking gels (125 mM Tris, pH 6.8) contained 5%, separating gels (750 mM Tris, pH 8.8) contained 12.5% acrylamide-bisacrylamide (37.5:1) solution, separation was performed at a constant voltage of 180 V in running buffer (25 mM Tris, 192 mM glycine, 0.1% w/v SDS, pH 8.3). Proteins were transferred to nitrocellulose membranes (Bio-Rad) in 25 mM Tris, 192 mM glycine, 20% v/v methanol, pH 8.4 transfer buffer. Blotting was performed at a constant current of 120 mA for 60 min. After blocking for 1 h in 20 mM Tris, 200 mM NaCl, 0.02% w/v NaN_3_, 5% w/v nonfat dry milk, pH 7.2 blotting buffer, membranes were incubated overnight at room temperature with MASP-1-SP-AP antibody or MASP-3 monoclonal antibody in the same buffer at 1 or 2 µg/ml, respectively. Membranes blotted with the MASP-3 antibody were washed (20 mM Tris, 200 mM NaCl, 0.02% w/v NaN_3_, 0.3% v/v Tween-20, pH 7.2) for 30 min, then incubated for 1 h at room temperature with alkaline phosphatase conjugated goat anti-mouse antibody at 2,000-fold dilution in blotting buffer. After washing for 45 min, membranes were visualized with NBT/BCIP solution (5 mM MgCl_2_, 100 mM Tris, 400 mg/l NBT, 200 mg/l BCIP, pH 9.0). Blots were scanned with an Epson Perfection 4490 scanner in 16-bit grayscale reflective mode. Densitometric analysis of the Western blots was performed using the Quantity One software (Bio-Rad).

### Determination of the MASP-3cf Activation Rate Constants by Various Proteases

Zymogen MASP-3cf at a final concentration of 2 µM (96 µg/ml) in 140 mM NaCl, 10 mM HEPES, pH 7.4, 0.1 mM EDTA buffer was incubated at 37°C alone (negative control), or in the presence of various proteases. The final concentrations of the tested proteases and the incubation times were as follows. Active MASP-1cf had been used previously at 100 nM (4.6 µg/ml), and samples were removed periodically for up to 5 h (11). Zymogen R448Q MASP-1cf was used at 1 µM (46 µg/ml), and samples were removed periodically for up to 13 days (the reaction was nearly complete at 6 days). Active MASP-2cf was used at 91 nM (3.9 µg/ml), and samples were removed periodically for up to 3 h, and a final overnight sample was also collected. Zymogen R444Q MASP-2cf was used at 1 µM, and samples were removed periodically for up to 6 days (no cleavage was detected). Active MASP-3cf (autocatalytic cleavage) was used at 500 nM, and samples were removed periodically for up to 5 days (no apparent increase in the amount of active MASP-3cf was detected). Zymogen MASP-3cf (self cleavage) as stated above was used at 2 µM (96 µg/ml), and samples were removed periodically for up to 6 days (no cleavage was detected). Active FD was used at 1 µM, and samples were removed periodically for up to 4 h (no cleavage was detected).

Samples were analyzed by SDS-PAGE under reducing conditions, followed by Coomassie Brilliant Blue G staining. Gels were scanned with an Epson Perfection 4490 scanner in 16-bit grayscale transparent mode. After scanning the intensities of the uncleaved substrate (zymogen MASP-3cf, 48 kDa) bands were quantified by densitometry using the BioRad Quantity One software. The intensities of the product (active MASP-3cf B chain, 31 kDa) bands were quantified in a similar fashion.

Substrate consumption was fitted using the *I*_S_ = *I*_B_ + *I*_o_ × exp(−*k*_obs_ × *t*) equation, where *I*_S_ stands for the intensity of the substrate band, *I*_o_ is the intensity of the substrate band at the zero time point, and *I*_B_ is the background intensity. When the substrate band overlapped with the enzyme band then the following equation was used instead: *I*_P_ = *I*_B_ + *I*_∞_ × [1 − exp(−*k*_obs_ × *t*)], where *I*_P_ is the intensity of the product band, and *I*_∞_ is the intensity of the product band at the completion of the reaction. First the observed pseudo first-order rate constants (*k*_obs_) were determined, then the *k*_obs_/[*E*]_T_ values were calculated, where [*E*]_T_ is the total enzyme concentration. The *k*_obs_/[*E*]_T_ value can be considered as an approximation of the catalytic efficiency (*k*_cat_/*K*_M_) according to the Michaelis–Menten kinetics if the substrate concentration is much less than the *K*_M_. The *k*_obs_/[*E*]_T_ values can also be used in kinetic simulations as approximate second-order rate constants.

### Kinetic Simulations

The fluid-phase activation and inhibition of lectin pathway proteases were modeled by kinetic simulations of the reaction network involving the zymogen, active, and inhibited forms of proteases and their inhibitors. The synthesis and elimination of the species were also included, resulting in a network of 25 reactions (with non-zero rate constants) between 10 species (Table S1 in Supplementary Material). The biochemical system simulator program COPASI version 4.19 (47) was used. Time course analysis was performed by solving the system of differential equations using the deterministic LSODA method (48).

## Results

### Isolation of a Mixture of MASPs from Blood

**Figure 1:**
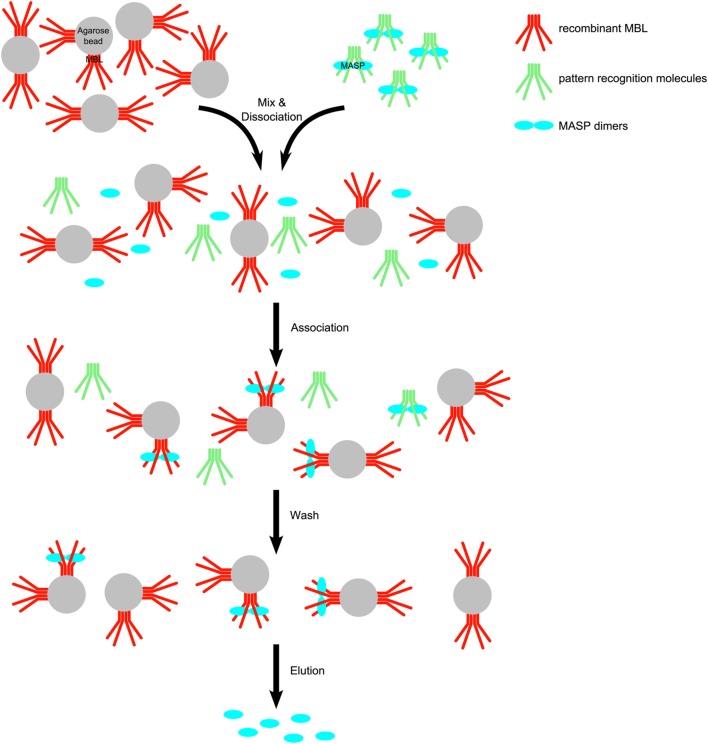
Scheme for the purification of mannose-binding lectin (MBL)-associated serine protease (MASPs) from human plasma. Human EDTA plasma was mixed with recombinant human mannan-binding lectin (rMBL)–Sepharose in the presence of high salt (1 M NaCl) causing to dissociate MASP– pattern recognition molecule (PRM) complexes. The ionic strength was decreased by dilution and excess CaCl_2_ was added to allow complexes to reassociate. MASPs (blue) are distributed between the original PRMs (green) from blood and the immobilized MBL (red). Soluble PRMs and soluble complexes are removed during the wash step. After washing, MASPs bound to rMBL-Sepharose were eluted with a buffer containing EDTA and high salt.

Recombinant human MBL was coupled to CNBr-Activated Sepharose as described in the Section “Materials and Methods.” Our goal was to determine the extent of MASP-3 activation under resting conditions. Thus, we purified a pool of MASPs from EDTA plasma, and in order to prevent further activation of zymogen MASP-3 by other proteases, we used inhibitors NPGB and Pefabloc during the whole process, and the purification was carried out at 0–8°C. We mixed MBL-Sepharose with plasma in a way that the estimated immobilized rMBL to plasma PRM ratio was about 3 to 1 by mass. At the first step in the presence of 1 M NaCl and 10 mM EDTA, the PRM-MASP complexes dissociated. Then the NaCl and EDTA concentrations were reduced by dilution and excess CaCl_2_ was added to initiate re-association of complexes. MASPs are expected to bind preferably to MBL-Sepharose as a result of the 3 to 1, immobilized rMBL to soluble PRM ratio. After reassociation the resin was packed into a column, and it was washed with low salt buffers to remove unbound proteins. The final elution buffer contained 1 M NaCl and 20 mM EDTA, dissociating MASPs (and MAPs) from the immobilized rMBL (Figure 1).

The samples were analyzed by SDS-PAGE followed by Western blotting. For detection our polyclonal MASP-1-specific antibody, or a commercial monoclonal MASP-3-specific antibody was used. Under reducing conditions, these antibodies recognize only the B-chain of active MASPs.

**Figure 2:**
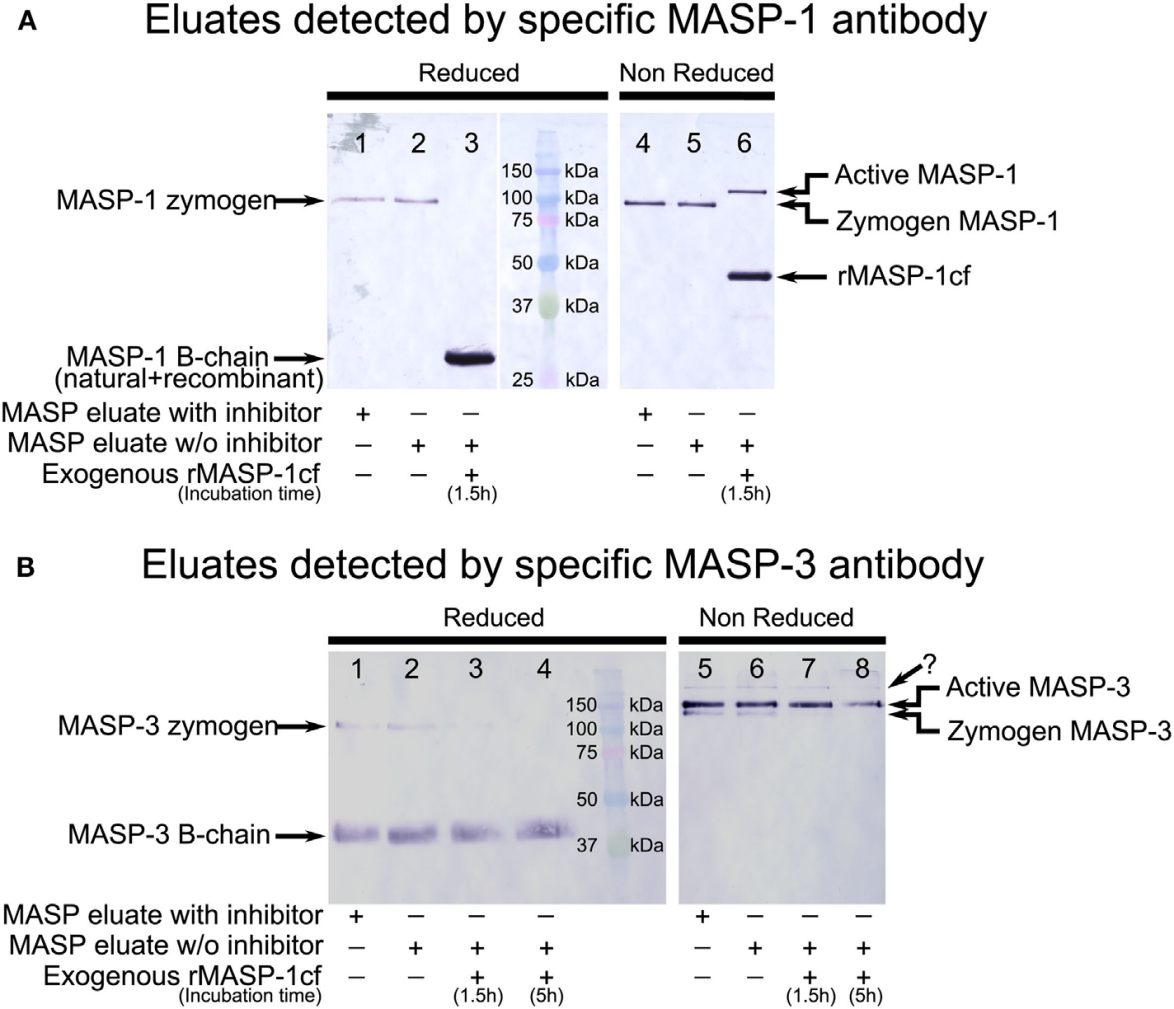
Western blot detection of mannose-binding lectin (MBL)-associated serine protease (MASP)-1 and MASP-3 isolated from normal human plasma in the presence of inhibitors. A pool of MASPs was purified from human plasma as outlined in Figure 1 in the presence of Pefabloc and 4-nitrophenyl 4′-guanidinobenzoate (NPGB) inhibitors. A sample was prepared in the same manner except that the inhibitors were omitted at the last elution step. Samples were analyzed by SDS-PAGE under reducing and non-reducing conditions followed by Western blotting and detected using a MASP-1- or a MASP-3-specific antibody. Both antibodies were developed against the serine protease (SP) domain of the corresponding protein, hence they can detect the whole molecule, or the B chain of the active form. **(A)** MASP-1 was present as a zymogen in the isolated samples running at about 95 kDa under reducing conditions. When exogenous active recombinant MASP-1cf was added to the inhibitor-free sample, plasma MASP-1 is converted to the active form. The B chains of plasma MASP-1 and MASP-1cf both ran at about 28 kDa under reducing conditions. Under non-reducing conditions, activation of plasma MASP-1 produced a slower-migrating band compared to the zymogen form. MASP-1cf runs at about 45 kDa under non-reducing conditions. **(B)** MASP-3 was present both in the zymogen and the active forms in the isolated samples. The zymogen ran at about 100 kDa under reducing conditions and the (glycosylated) B-chain of the active form ran at about 40 kDa. Under non-reducing conditions active MASP-3 migrated slower than the zymogen form. The faint band, indicated by a question mark, running above the active form is probably due to non-specific binding. Addition of active recombinant MASP-1cf to the inhibitor-free sample caused the disappearance of the MASP-3 zymogen bands.

In order to identify the bands corresponding to the active and the zymogen variants under non-reducing conditions, we also prepared samples containing neither NPGB nor Pefabloc. Only the last washing and the elution buffers lacked the inhibitors. The eluate was incubated with MASP-1cf, and samples were withdrawn at 1.5 and 5 h. Upon this treatment under reducing conditions the bands corresponding to zymogen MASP-1 and MASP-3 disappeared, and only the B-chains are seen, as expected. Under non-reducing conditions upon addition of MASP-1cf, the faster migrating bands disappeared, and only the slower migrating bands remained. Hence, the faster migrating band corresponds to the zymogen form, and the slower migrating band corresponds to the active form using non-reduced samples under our SDS-PAGE conditions (Figure 2).

### MASP-3 Is Mostly Active, Whereas All MASP-1 Is Zymogenic in the Same Isolates

During the isolation, we used NPGB and Pefabloc SC to prevent activation of zymogen MASP-3 by any protease. Under non-reducing conditions zymogen and active MASP-3 are well separated by SDS-PAGE, on the other hand the mobilities of the two forms are only slightly different, which is favorable for the quantification.

For the quantification of the two forms of plasma MASP-3, we had several reasonable assumptions as follows: (I) the MBL-Sepharose resin binds zymogen and active MASP-3 with the same affinity, and no further activation occurs during the isolation; (II) the transfer efficiencies during Western blotting are equivalent due to the small difference between the mobility of zymogen and active MASP-3 under non-reducing conditions; and (III) the commercial monoclonal MASP-3-specific antibody recognizes both forms of MASP-3 with the same sensitivity.

First, we measured the activation state of plasma MASP-1 in our isolates as a control. We found that all MASP-1 remained zymogenic during the preparation, proving that the lectin pathway did not activate during the isolation (Figure 2A, lanes 1–2 and 4–5). It is in agreement with previous articles showing that MASP-1 is found in the zymogen form in the circulation (49, 50).

**Figure 3:**
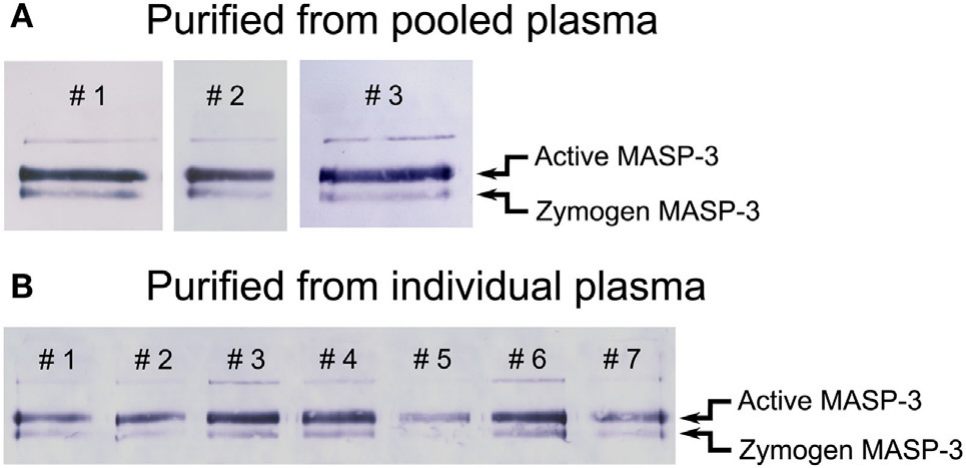
Western blot analysis of purified mannose-binding lectin (MBL)-associated serine protease (MASP) pools detected by a MASP-3-specific antibody. MASPs were purified from human plasma as outlined in Figure 1 in the presence of Pefabloc and 4-nitrophenyl 4′-guanidinobenzoate (NPGB). Samples were analyzed by SDS-PAGE under non-reducing conditions followed by Western blotting and detection using a MASP-3-specific antibody. The faint band running above the active form is probably due to non-specific binding of the antibody. Western blots were quantified as described in the Section “Materials and Methods” and the result are listed in Table 1. **(A)** The analysis of three parallel preparations starting from the same pool of human EDTA plasma. **(B)** The analysis of samples purified from the plasma of seven individuals. The full blots are provided as Figure S1 in Supplementary Material.

In contrast, in the same isolates we detected both forms of MASP-3 (Figure 2B, lanes 1–2, 5–6). We performed additional three parallel isolations with pooled plasma, and each blot is shown in Figure 3A. Our results indicate that MASP-3 circulates mostly in the active form, and quantification revealed that the active–zymogen ratio was about 4 to 1 (Table 1).

**Table 1:** The fraction of active MASP-3 from pooled and individual plasma samples.

Source sample	Fraction of active MASP-3 (%)
Pooled plasma	78 ± 5
Individual plasma #1	76 ± 5
Individual plasma #2	79 ± 7
Individual plasma #3	86 ± 2
Individual plasma #4	83 ± 2
Individual plasma #5	85 ± 5
Individual plasma #6	76 ± 3
Individual plasma #7	79 ± 3
Average of the individual samples	81 ± 4^a^

*Average ± SD from at least three parallels. The pooled plasma value represents average from three independent isolations, whereas the individual samples were run three times using the same isolate*.

*^a^Average and deviation of the averages*.

### Low Individual Variation of the Active–Zymogen Ratio of MASP-3

In order to assess the individual variation of the active to zymogen ratio for MASP-3, we examined EDTA plasma samples from seven healthy donors. The isolation procedure of MASPs from the individual plasma samples was the same as from the pooled samples. Each sample was analyzed three times by non-reducing SDS-PAGE followed by Western blot, then quantified. Figure 3B represents all individual samples together on the same blot membrane. In every sample MASP-3 was over 70% activated. While the total MASP-3 level can be quite different in each donor (36, 37), the active to zymogen ratio showed relatively low variation (Table 1).

### *In Vitro* Activation of MASP-3 by Various Complement Proteases

Zymogen MASP-1 and MASP-2 are able to undergo autoactivation, and the active MASPs can activate their proenzymic counterparts (41, 42), although for MASP-2 the autoactivation process is much slower (11). In contrast, Zundel et al. showed that neither the zymogen nor the active form of MASP-3 has a similar activity at all (46). We observed the same using the catalytic fragments of MASP-3 (Table 2).

**Table 2:** Rate constants for the activation of zymogen MASP-3cf.

Enzyme	*k*_obs_/[*E*]_T_ (M^–1^ s^–1^)
Active MASP-1cf	1.2 ± 0.1 × 10^3a^
Zymogen R448Q MASP-1cf	3.8 ± 0.9^b^
Active MASP-2cf	2.7 ± 0.5 × 10^3b^
Zymogen R444Q MASP-2cf	~0
Active MASP-3cf	~0^c,d^
Zymogen MASP-3cf	~0^d^
Active FD	~0

*The *k*_obs_/[*E*]_T_ value can be considered as an estimate for the catalytic efficiency (*k*_cat_/*K*_M_). ~0 indicates that no significant cleavage occurs even after several days*.

*^a^From Megyeri et al. (11)*.

*^b^Average ± SD, n = 4 is indicated*.

*^c^A very slow cleavage may occur corresponding to a *k*_obs_/[*E*]_T_ << 1 M^–1^ s^–1^*.

*^d^Originally observed by Zundel et al. (46) on full-length MASP-3 variants and confirmed by us using the catalytic fragments*.

It is now generally accepted that MASP-1, after autoactivation, activates zymogen MASP-2 (9–11). *In vitro*, active MASP-1 is able to activate MASP-3 as well (10, 21), and the cleavage rate has been determined using the catalytic fragments (11).

**Figure 4:**
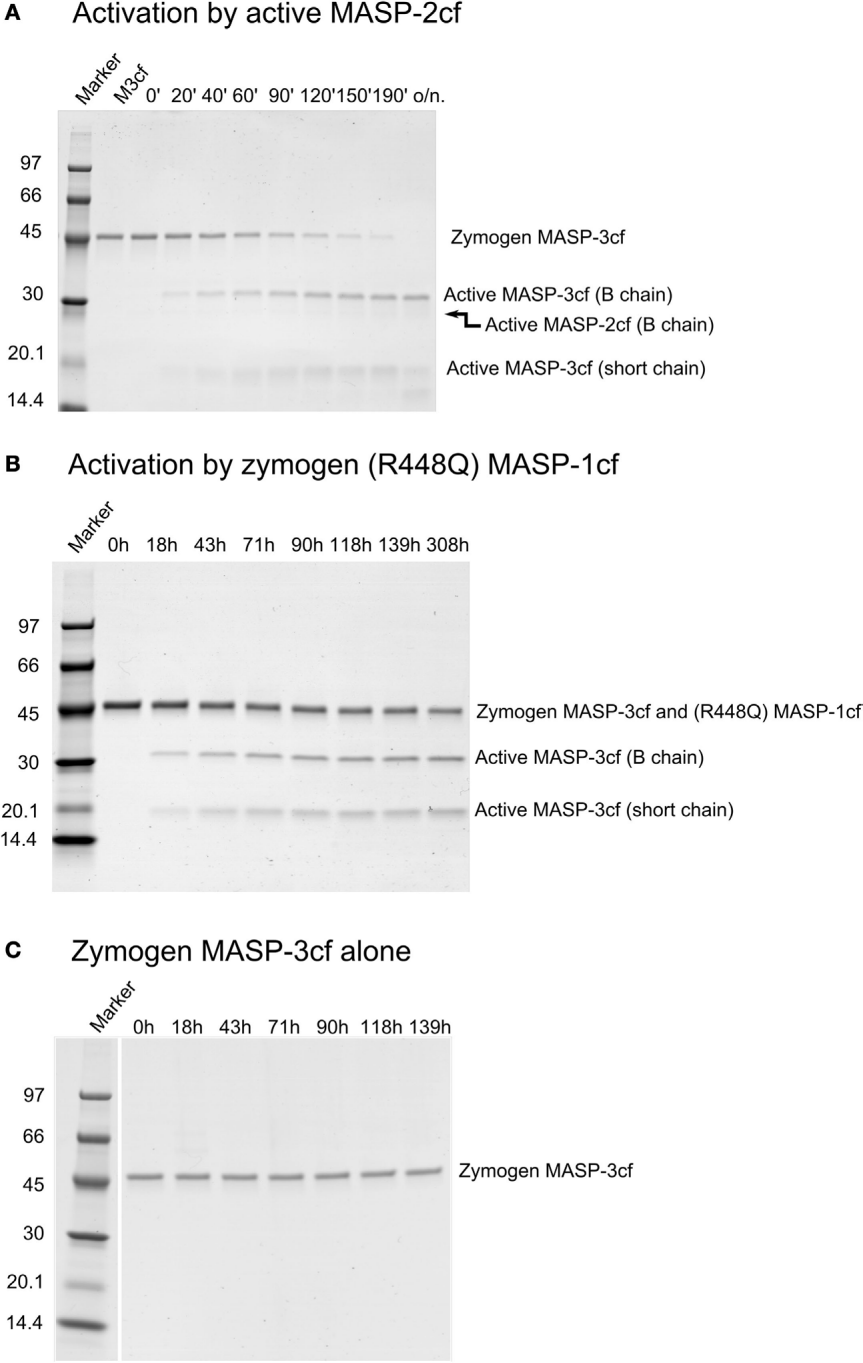
Activation of zymogen mannose-binding lectin (MBL)-associated serine protease (MASP)-3cf. Zymogen MASP-3cf at 2 µM was incubated with active MASP-2cf, or zymogen R448Q MASP-1cf, or alone. Aliquots were removed periodically at time points as indicated. Samples were analyzed by SDS-PAGE under reducing conditions. Molecular weights of the marker proteins in kDa are indicated. Zymogen MASP-3cf runs at about 48 kDa, while the active form gives two bands at about 31 and 17 kDa. Representative gels are shown as examples. **(A)**. Activation by 91 nM active MASP-2cf. The band corresponding to the B chain of MASP-2cf (about 27 kDa) is very faint due to its low concentration. Lane 2 had zymogen MASP-3cf (M3cf) alone. **(B)** Activation by 1 µM zymogen R448Q MASP-1cf. The band of zymogen R448Q MASP-1cf (about 46 kDa) comigrated wit that of zymogen MASP-3cf. Quantification was carried out as described in the Section “Materials and Methods” and the determined rate constant are listed in Table 2. **(C)** Zymogen MASP-3cf alone is shown as a control demonstrating that it does not autoactivate or cleaved by any potential contaminant upon prolonged incubation.

For this study, we determined the missing rate constants of potential reactions that can produce active MASP-3 using the catalytic fragments of MASPs (Table 2; Figure 4). Out of curiosity we checked weather FD can activate MASP-3cf, but we could not detect any cleavage (data not shown, Table 2).

Similarly to active MASP-1cf, active MASP-2cf can also activate zymogen MASP-3cf at an approximately two-fold higher a rate (Figure 4A). It should be noted, however, that in the blood MASP-2 has a 20-fold lower concentration compared to MASP-1, hence its contribution to MASP-3 activation is likely to be much less than that of MASP-1 *in vivo*. Zymogen MASP-2cf R444Q had no MASP-3cf cleaving activity at all (data not shown, Table 2). Interestingly the stable zymogen R448Q variant of MASP-1cf was also capable to activate MASP-3cf with a 300-fold lower rate compared to the active enzyme (Figure 4B). Although this reaction is rather slow, it might have a significance as the major variant of MASP-1 in resting blood is the zymogen form.

At the same time, zymogen MASP-3cf, when incubated alone, did not show any sign of activation upon prolonged incubation (Figure 4C), demonstrating that it does not autoactivate, and it is not contaminated by any external activating proteases. Also, no significant cleavage of zymogen MASP-3cf by active MASP-3cf was observed (data not shown, Table 2).

### *In Silico* Modeling of the Fluid Phase Basal Activation of Lectin Pathway Proteases

We have determined previously (11, 12, 51) and for this study (Table 2) essentially all the possible activation rate constants between lectin pathway proteases using the catalytic fragments. These reactions include zymogen autoactivation, cross-activation between the zymogen forms, autocatalytic activation, and cross-activation of a zymogen by another active MASP. Altogether there are 3 × 3 × 2 = 18 possible reactions, however “only” 10 have non-zero rate constants and “only” 9 have a significant value (Table S1 in Supplementary Material). Activated MASP-1 and MASP-2 are constantly inactivated by C1 inhibitor and antithrombin (12, 51). These reactions were also added to the reaction network (Table S1 in Supplementary Material). C1 inhibitor might reversibly inhibit zymogen MASP-1 and MASP-2 (42), however this possible effect was not included into the model, because no quantitative data are available for these interactions. Proteins are continuously synthesized and eliminated from the circulation. There are no data for human MASPs in this regard, however in mice the half life of MASP-1 and MASP-3 is about 1.5 h (52). In our model, we used 6 h (arbitrarily) for all MASPs as humans have slower metabolism. This value corresponds to a 3.31 × 10^−5^ s^−1^ first order elimination rate constant. The same value was used for all forms of MASPs including the serpin inhibited forms. This is a valid assumption as it was shown for C1s and C1s-C1 inhibitor complex in guinea pig (53). Synthesis rates (Table S1 in Supplementary Material) were calculated from the elimination rate constants and the measured steady-state plasma concentrations of MASPs (39).

**Figure 5:**
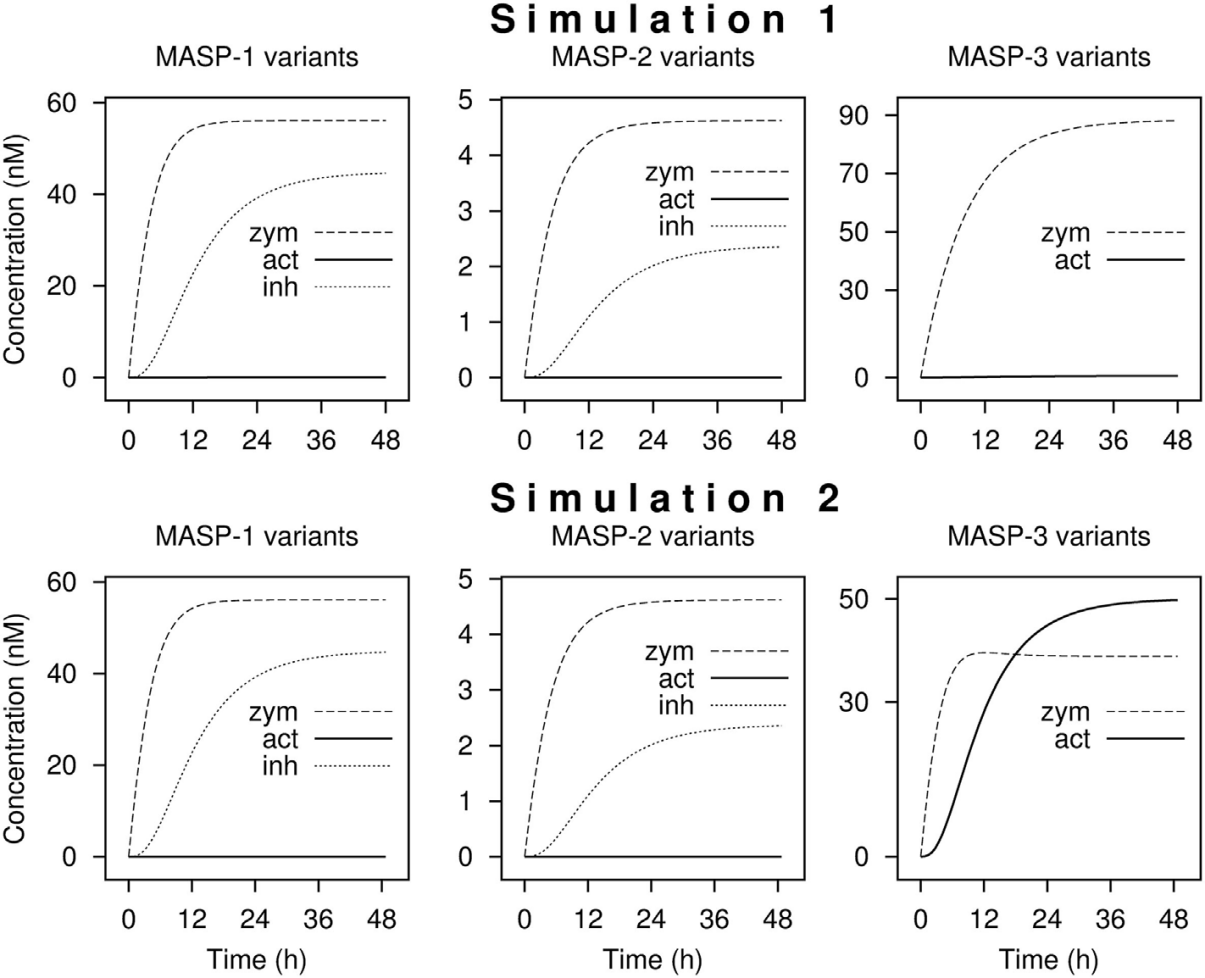
Kinetic simulations of the activation of lectin pathway proteases. Time courses of the concentrations of zymogen (zym), active (act), and combined C1-inhibitor and antithrombin bound (inh) forms of MASP proteases from kinetic simulations are shown starting with zero levels of mannose-binding lectin (MBL)-associated serine protease (MASPs). Simulation 1 was performed with measured rate constants. For Simulation 2, the rate constant of the activation of MASP-3 by zymogen MASP-1 was increased by a factor of 200. The full set of kinetic parameters is found in Table S1 in Supplementary Material. The reaction set is depicted in Figure 6 within the boxed area. Steady-state distributions are shown in Table 3.

Including the 10 activation, 4 inactivation, 3 synthesis, and 8 elimination reactions a network of 25 reactions was set up. Starting with zero MASP concentrations, and assuming that MASPs are synthesized as proenzymes, simulations were run until steady-state concentrations were obtained. Simulation 1 was run using the measured activation rate constants (Figure 5 upper panels). According to simulation 1, only very little active MASP-3 is attained at steady-state (Table 3), which is not in line with our data. However, the simulation is in line with the observations that MASP-1 and MASP-2 circulate mostly as zymogens (49), and some of them circulate as serpin-inhibited complexes (54, 55). It must be noted that our simulation cannot predict the precise ratios of the different MASP forms, because several simplifications were used, however, it is suitable to predict the general trends.

**Table 3:** Distribution of zymogen, active, and serpin-inhibited forms of MASP molecules in the steady-state from kinetic simulations.

Molecule	Variant	Fraction (%)	
		Simulation 1	Simulation 2
MASP-1	Zymogen	55.7	55.7
	Active	0.016	0.016
	Inhibited	44.3	44.3

MASP-2	Zymogen	66.3	66.2
	Active	0.00042	0.00042
	Inhibited	33.7	33.8

MASP-3	Zymogen	99.3	43.8
	Active	0.68	56.2

*Simulation 1 was performed with measured rate constants. For simulation 2, the rate constant of MASP-3 activation by zymogen MASP-1 was increased by a factor of 200*.

If we assume that a fraction of MASP-1 and MASP-3 reside on the same complexes and efficient intracomplex activation between zymogen MASP-1 and zymogen MASP-3 occurs, this would correspond to a much faster reaction than that between isolated molecules. To account for this scenario the rate constant of MASP-3 activation by zymogen MASP-1 was increased arbitrarily by a factor of 200 (Simulation 2, Figure 5 lower panels). By this single modification, the active MASP-3 steady-state fraction increased to over 50% (Table 3), which is more in line with the observed >70% value. Values for MASP-1 and MASP-2 variants remained essentially the same. Of course this scenario raises several questions, which are discussed later.

### Comparison of the Activation Loop Sequences of MASPs, C1r, and C1s

The activation loop of MASP-3 has an unusual sequence in comparison with those of MASP-1, MASP-2, C1r, and C1s (Table 4). The activation site is a R-I bond, which is the same in all the listed proteases. However, in MASP-3 the P1 Arg residue is preceded by a Lys residue, giving rise to a paired basic (KR) motif preceding the activation site. This motif coincides with the recognition motif of basic amino acid-specific proprotein convertases, which is (K/R) − (X)*_n_* − (K/R)↓, where *n* = 0, 2, 4, or 6 and X is any amino acid (56). Most often the cleavage site is after consecutive basic residues. There are of course other segments in the listed proteases, where a similar motif can be found, however the activation site of MASP-3 is exposed and hence suitable for such cleavage. The possibility of MASP-3 activation by proprotein convertases is also discussed later.

**Table 4:** P6-P6′ sequences of MASPs, C1r, and C1s.

Protease	P6	P5	P4	P3	P2	P1	P1′	P2′	P3′	P4′	P5′	P6′
MASP-1	R	K	L	M	A	**R**	**I**	F	N	G	R	P
MASP-2	R	T	T	G	G	**R**	**I**	Y	G	G	Q	K
MASP-3	P	S	L	V	**K**	**R**	**I**	I	G	G	R	N
C1r	V	E	Q	R	Q	**R**	**I**	I	G	G	Q	K
C1s	F	E	E	K	Q	**R**	**I**	I	G	G	S	D

*Bold highlights the scissile R-I bond, whereas the potential recognition site (KR) for proprotein convertases is underlined*.

## Discussion

Our previous studies revealed that active MASP-3 is the exclusive pro-FD activator in resting human blood (22, 23). This implied that active MASP-3 must be present in the blood, even without ongoing complement activation above the baseline level. We decided to confirm this assumption by a different approach, and to quantify the extent of MASP-3 activation, i.e., the active to zymogen ratio.

Initially, we attempted to detect MASP-3 directly in plasma samples, but the available antibodies were not suitable for this purpose because of the very high background. Therefore, we decided to enrich our samples for MASP-3. A mixture of MASPs and MAps was purified from EDTA plasma using MBL-Sepharose as described in the presence of broad specificity SP inhibitors in order to avoid any activation during the isolation. We detected MASP-3, and as a control MASP-1, by Western blotting in the purified samples. For quantification, the non-reduced samples are more suitable, because the two chains of active MASP-3 are held together by a disulfide bond, hence the masses of the active and zymogen forms are virtually identical, and their mobilities in polyacrylamide gels are very similar, though still allow their separation.

Mannose-binding lectin-associated SP-1 was always proenzymic, whereas MASP-3 was a mixture of active and zymogen in all tested samples with active MASP-3 being the dominant form. On average, we found that MASP-3 was about 80% active, and in individual samples it was always more than 70% active, displaying little individual variation. Using another technique, i.e., using anti-MASP-3 to pull out MASP-3, using different enzyme inhibitors and using different anti-MASP-3 antibodies on the Western blot also leads to the finding that a high proportion of the MASP-3 is found as activated MASP-3 (25).

We and others have postulated before that active MASP-1 and MASP-2 is capable of activating MASP-3 (10, 11, 21). The rate constant for active MASP-1cf was measured previously (11), and here we also measured the activation rate constant for active MASP-2cf, which activates MASP-3cf about twice as fast as active MASP-1cf. It occurred to us that FD might reciprocally activate MASP-3, however, FD did not cleave zymogen MASP-3cf, neither did zymogen R444Q MASP-2cf. The zymogen MASP-1cf (R448Q variant) activated MASP-3cf slowly, but the rate constant was about 300-fold lower than that for active MASP-1cf.

Isolated full-length recombinant human MASP-3 does not autoactivate (46), neither does the catalytic fragment as previously shown (57) and unambiguously proved here using highly purified MASP-3cf. It is notable that recombinant MASP-3 can undergo slow activation upon prolonged storage. Zundel et al. (46) observed this cleavage with the wild-type and the S645A (precursor numbering S664A) inactive variant of MASP-3 as well, and concluded that the observed activation is due to cleavage by a contaminating protease from the cell culture supernatant. Similar observations were made by Pihl et al. (25). These observations indicate that the scissile R-I (449–450 precursor numbering) bond is sensitive to proteolysis by other proteases in general. Iwaki et al. (21) detected activation of recombinant mouse MASP-3 (also produced as proenzyme) in complex with MBL-A on *Staphylococcus aureus*. The authors postulated it as self-activation, but it is possible that a protease from *S. aureus* activated MASP-3. No activation was observed on uncomplexed MASP-3, MASP-3 in complex with other PRMs, or when other activators like mannan were used (21). In all, data in the literature with full-length dimeric MASP-3 and our observations with monomeric catalytic fragments indicate that MASP-3 does not autoactivate in general, and most importantly no autoactivation of MASP-3 was observed in the fluid phase.

**Figure 6:**
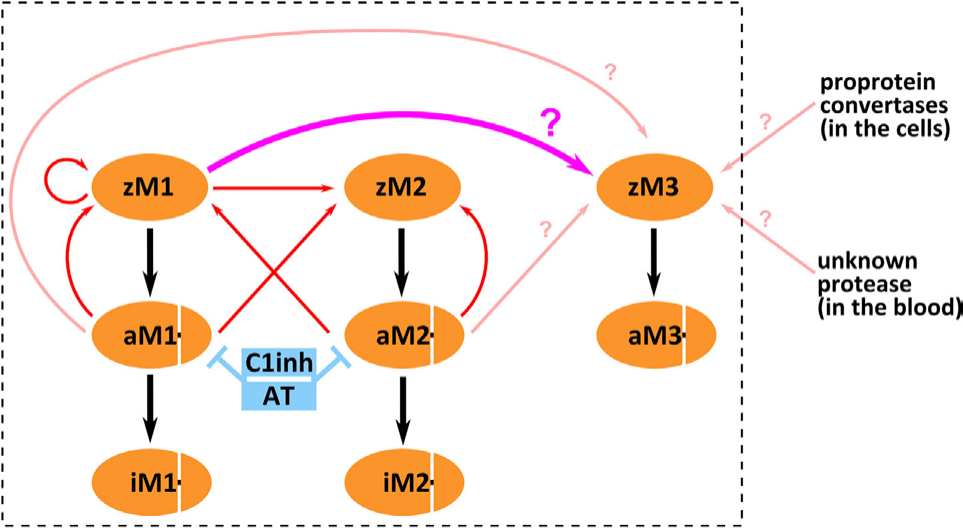
Possible mechanisms of mannose-binding lectin (MBL)-associated serine protease (MASP)-3 activation. The boxed part depicts activation and inactivation steps involving only lectin pathway proteases. Three forms of MASP-1 (zM1 for zymogen MASP-1, aM1 for active MASP-1, and iM1 for serpin-inhibited MASP-1), three forms of MASP-2 (zM2 for zymogen MASP-2, aM2 for active MASP-2, and iM2 for serpin-inhibited MASP-2), and two forms of MASP-3 (zM3 for zymogen MASP-3 and aM3 for active MASP-3) are shown as separate variants. Red arrows pointing from the enzyme to the substrate represent previously established activation reactions involving MASP-1 and MASP-2. Pink arrows (pointing from the enzyme to the substrate) represent possible activation reactions producing active MASP-3. Cleavage of zymogen MASP-3 by zymogen MASP-1 is highlighted by thick pink arrow (again pointing from the enzyme to the substrate). Inhibition by serpins is indicated by blue T-shaped symbols. Black arrows simply indicate conversion.

It is enigmatic that MASP-3, which does not autoactivate, is present mostly in the active form in the circulation, whereas MASP-1, which has a potent autoactivation capacity, is proenzymic. MASP-3 must be activated obviously by another protease, or proteases. Its activator may be another lectin pathway protease, or a yet unidentified protease in the blood, or possibly it is activated before secretion within the cells (Figure 6). In order to assess the first possibility, we set up a kinetic model for the fluid-phase basal level activation of lectin pathway proteases using available and newly determined kinetic constants.

Previously, we have determined the rate constants for all possible activation steps involving MASP-1 and MASP-2 using the catalytic fragments (11). In this study, we added the missing rate constant (see above) for reactions producing active MASP-3. MASP-1 and MASP-2 are known to be inhibited by C1 inhibitor and antithrombin (4, 58, 59), while MASP-3 is not (46, 60). Rate constants for the inactivation reactions had been previously determined (12, 51) and they were included into the model. Estimated elimination and synthesis rates were also included.

Running the simulation with the determined rate constant does not explain the observed phenomenon, i.e., that MASP-3 is present mostly in the active form. It must be noted, however, that our kinetic model can adequately describe intermolecular reactions, but it is less suitable to describe intramolecular (intracomplex) cleavage reactions.

We have attempted to modify the kinetic parameters to account for a potentially more efficient intramolecular (intracomplex) activation. By changing just one parameter—the activation reaction of MASP-3 by zymogen MASP-1 was arbitrarily increased by 200-fold—the output became similar to the observed facts that MASP-2 and MASP-1 are proenzymic, and MASP-3 is extensively activated. Moreover, the presence of serpin-inhibited MASP-1 and MASP-2 complexes was observed in blood samples by others (54, 55) even in resting blood, and our simulations are completely in line with these observations.

It may occur to someone that if MASP-3 can be activated efficiently by zymogen MASP-1 intramolecularly within a complex, why would not all zymogen MASP-1 autoactivate in a similar way, which is potentially a faster reaction. This question leads to a more general problem, i.e., how the different lectin pathway complexes really look like, and how they are really activated. This is a difficult question, and several activation scenarios have been proposed (61–63). It is possible that MASP-3 is only activated on highly multimeric PRMs that can accommodate dimers of both MASP-1 and MASP-3.

There are alternative explanations to account for the presence of active MASP-3 in circulation. It was proposed earlier for FD (27) that it might be activated within the secretory pathway in mammalian cells. The N-terminal sequence of pro-FD, APPRGR↓, however does not match the general recognition sequence of basic amino acid-specific proprotein convertases, which is (K/R) − (X)*_n_* − (K/R)↓, where *n* = 0, 2, 4, or 6 (56). In contrast, MASP-3 contains a paired basic residue motif at the activation site (SLVKR↓I), which satisfies the above-mentioned criterion. The observation that recombinant MASP-3 was always expressed as a proenzyme (21, 37, 46) even in mammalian cells contradicts this hypothesis. Therefore, if MASP-3 is activated intracellularly, it must be cell-type specific. Supporting this hypothesis is that furin and PACE4 can activate MASP-3 *in vitro* (64).

In summary, we have demonstrated that MASP-3, just like FD, circulates preferably in the active form in resting human blood. It is now clear that activated MASP-3 keeps FD mostly activated in the circulation, even in the absence of any complement triggering factors. Both active MASP-1 and active MASP-2 are able to activate MASP-3 fairly well. This mechanism might be important during infections. However, both MASP-1 and -2 are present as proenzymes in resting blood. The enigma cannot be easily resolved, but the ability of zymogen MASP-1 to cleave zymogen MASP-3 may provide a solution. We set up a kinetic model for the activation of lectin pathway proteases, which, along with other activation scenarios for MASP-3, is summarized in Figure 6. Our kinetic model, which also includes inactivation by serpins, adequately explains why MASP-1 and MASP-2 are proenzymic, but it can explain extensive MASP-3 activation only if we assume efficient intracomplex activation by zymogen MASP-1. Our kinetic model provides a reasonable explanation, however, elucidating the physiological activation mechanism of MASP-3 requires further experiments.

## Ethics Statement

The study was conducted in conformity with the WMA Declaration of Helsinki. Experimental protocols were approved by the local ethics committee (permission number: TUKEB 9190-1/2017/EKU). Informed consent was obtained for the isolation of peripheral venous blood from the donors.

## Author Contributions

JD designed the study. GO and JD wrote the article. GO, RD, and JD performed the experiments. AS performed the kinetic simulations. ST provided recombinant human MBL. JD, GO, PZ, and PG analyzed the data. All authors revised and approved the manuscript.

## Conflict of Interest Statement

The authors declare that the research was conducted in the absence of any commercial or financial relationships that could be construed as a potential conflict of interest.

## Abbreviations

MBL, mannose-binding lectin; MASP, MBL-associated serine protease; cf, catalytic fragment; MAp, MBL-associated protein; PRM, pattern recognition molecule; CL, collectin; SP, serine protease; CUB, C1r/C1s, sea urchin Uegf and bone morphogenetic protein-1; EGF, epidermal growth factor; CCP, complement control protein; FD, factor D; pro-FD, proenzyme factor D; AP, alkaline phosphatase; 3MC, Malpuech–Michels–Mingarelli–Carnevale; NPGB, 4-nitrophenyl 4′-guanidinobenzoate.
