# Supplementary material for: Extensive Basal Level Activation of Complement Mannose-Binding Lectin-Associated Serine Protease-3: Kinetic Modeling of Lectin Pathway Activation Provides Possible Mechanism
Source: Front Immunol. 2017 Dec 18;8:1821. doi: 10.3389/fimmu.2017.01821 (PMC5741598; doi:10.3389/fimmu.2017.01821)
Supplement: Supplementary file 1 [file table_1.PDF]

Supplementary TABLE 1. The full set of parameters used for the kinetic simulations of fluid-phase activation and subsequent inhibition reactions of lectin pathway proteases

Enzyme	Substrate	Product	Order	k or v	Unit	Notes
zM1	zM1	aM1 (+ zM1)	2	4.5×10^{2}	$M^{-1}\ s^{-1}$	zymogen autoactivation
aM1	zM1	aM1 (+ aM1)		8.3×10^{4}		autocatalytic activation
zM1	zM2	aM2 (+zM1)		3.0×10^{2}		
aM1	zM2	aM2 (+aM1)		1.2×10^{4}		
**zM1**	**zM3**	**aM3 (+zM1)**		**3.8**		increased 200-fold in simulation 2
**aM1**	**zM3**	**aM3 (+aM1)**		$\mathbf{1.2 \times 10^{3}}$		
zM2	zM2	aM2 (+zM2)	2	0.14	$M^{-1}\ s^{-1}$	zymogen autoactivation (the measured 0.14 $M^{-1}\ s^{-1}$ is negligible)
aM2	zM2	aM2 (+aM2)		6.0×10^{2}		autocatalytic activation
zM2	zM1	aM1 (+zM2)		0		
aM2	zM1	aM1 (+aM2)		4.1×10^{3}		
**zM2**	**zM3**	**aM3 (+zM2)**		**0**		
**aM2**	**zM3**	**aM3 (+aM2)**		$\mathbf{2.7 \times 10^{3}}$		
**zM3**	**zM3**	**aM3 (+zM3)**	2	**0**	$M^{-1}\ s^{-1}$	zymogen autoactivation
**aM3**	**zM3**	**aM3 (+aM3)**		**~0**		autocatalytic activation
zM3	zM1	aM1 (+zM3)		0		assumed to be negligible
aM3	zM1	aM1 (+aM3)		0		
zM3	zM2	aM2 (+zM3)		0		
aM3	zM2	aM2 (+aM3)		0		
aM1	C1i	iM1	2	6.7×10^{3}	$M^{-1}\ s^{-1}$	measured in the presence of heparin
aM2	C1i	iM2		1.3×10^{6}		
aM1	AT	iM1		4.0×10^{4}		
aM2	AT	iM2		4.7×10^{4}		
-	-	zM1	0	3.34×10^{-12}	$M\ s^{-1}$	synthesis rate
-	zM1	-	1	3.31×10^{-5}	s^{-1}	elimination
-	aM1	-		3.31×10^{-5}		
-	iM1	-		3.31×10^{-5}		
-	-	zM2	0	2.32×10^{-13}	$M\ s^{-1}$	synthesis rate
-	zM2	-	1	3.31×10^{-5}	s^{-1}	elimination
-	aM2	-		3.31×10^{-5}		
-	iM2	-		3.31×10^{-5}		
-	-	zM3	0	2.94×10^{-12}	$M\ s^{-1}$	synthesis rate
-	zM3	-	1	3.31×10^{-5}	s^{-1}	elimination
-	aM3	-		3.31×10^{-5}		

Reactions that can theoretically produce active MASP-3 are indicated by **bolding**. Among these **blue** color indicates reactions with non-zero rate constant.

Abbreviations:
zM1 = zymogen MASP-1, aM1 = active MASP-1, iM1 = MASP-1 / serpin complexes,
zM2 = zymogen MASP-2, aM2 = active MASP-2, iM2 = MASP-2 / serpin complexes,
zM3 = zymogen MASP-3, aM3 = active MASP-3
C1i = C1 inhibitor, AT = antithrombin

Assuming a half-life of $t_{1/2}$ = 6 h the elimination rate constant is: $k_{elim} = \ln 2 / t_{1/2} = 3.31 \times 10^{-5}\ s^{-1}$
Assuming a steady-state "c" level the synthesis rate is calculated as: $v_{syn} = c \times k_{elim}$

The used steady-state concentrations:
c_{M1} = [zM1] + [aM1] + [iM1] = 101 nM
c_{M2} = [zM2] + [aM2] + [iM2] = 7 nM
c_{M3} = [zM3] + [aM3] = 89 nM
[C1i] and [AT] were each set to a 2000 nM constant value
