# Supplementary material for: Extensive Basal Level Activation of Complement Mannose-Binding Lectin-Associated Serine Protease-3: Kinetic Modeling of Lectin Pathway Activation Provides Possible Mechanism
Source: Front Immunol. 2017 Dec 18;8:1821. doi: 10.3389/fimmu.2017.01821 (PMC5741598; doi:10.3389/fimmu.2017.01821)

Supplementary FIGURE 1 | Western blot analysis of purified MASP pools detected by a MASP-3 specific antibody. This figure shows the full length blots depicted on **Figure 3**. MASPs were purified from human plasma as outlined in **Figure 1** in the presence of Pefabloc and NPGB. Samples were analyzed by SDS-PAGE under non-reducing conditions followed by Western blotting and detection using a MASP-3 specific antibody. The faint bands in certain lanes running above the active form, and below the zymogen form are probably due to non-specific binding of the antibody. The marker (molecular weight indicated in kDa) was run under reducing conditions, hence the analyzed non-reduced samples are separated by an empty lane. MASP-3 has a molecular weight of about 100 kDa, however non-reduced MASP-3 migrated about as fast as the 150 kDa reduced marker protein. Western blots were quantified as described in the Materials and Methods and the result are listed in **Table 1**. **(A)** The analysis of three parallel preparations starting from the same pool of human EDTA plasma. **(B)** The analysis of samples purified from the plasma of seven individuals.

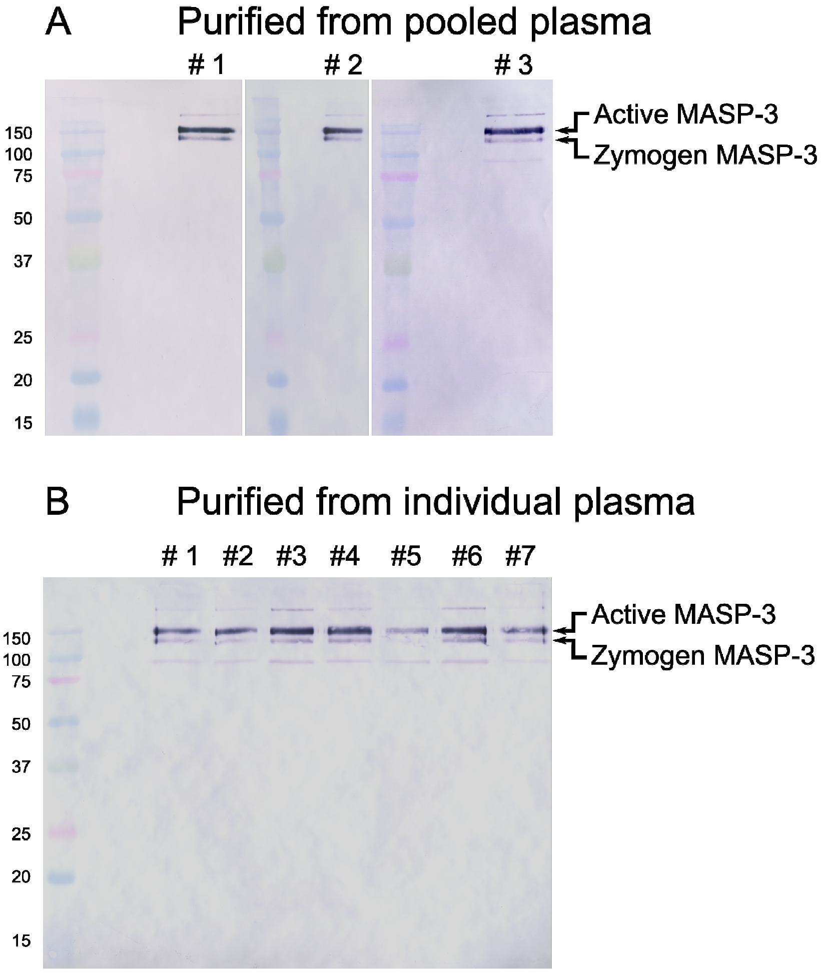

Supplement: Supplementary file 2 [file image_1.PDF]
